# New York Academy of Medicine

**Published:** 1863-08

**Authors:** James Anderson

**Affiliations:** President, in the Chair


### MISCELLANEOUS.

### NEW YORK ACADEMY OF MEDICINE.

*Stated Meeting, July* 1, 1863.

DR. JAMES ANDERSON, PRESIDENT, IN THE CHAIR.

### *Alcoholic Stimulants in Pulmonary Tuberculosis—Discussion of Dr. Flint's Paper.*

Dr. Flint read an elaborate and interesting paper, entitled the "*Management of Pulmonary Tuberculosis, with Special Reference to the Employment of Alcoholic Stimulants.*" It consisted mainly of a clinical report, based upon sixty-two cases of arrested tuberculosis. These cases are analysed and compared as regards points of agreement relating directly and indirectly to the management, the main objects of inquiry being the evidence afforded of self-limitation, the influence of hygienic measures, the agency of remedies, and the importance of alcoholic stimulants in determining the arrest of the disease. He considers that the disease is arrested whenever the general and local symptoms show it to be non-progressive for several consecutive months. After the arrest, the recovery may or may not be complete. In many of the cases the recovery was complete, while in others a certain amount of cough and expectoration continued for a considerable period of time, in two cases for more than twenty, and in one case for forty years.

For convenience of analysis he arranges the cases in three groups.—I—Those in which no curative or hygienic methods of management were employed. II.—Those cases in which hygienic measures were employed. III.—Cases in which remedial measures, including alcoholic stimulants, were supposed to have had a curative influence. I.—In the first group seven cases are collected, of which four recovered entirely. II.—The second group includes twenty cases, in twelve of which the recovery appeared to be complete, in eight the arrest of the disease was not followed by complete recovery within the period that the condition of the patients severally was known. The ages in this group ranged between nineteen and fifty years, and seventeen of these were males. In only four of the cases are there any grounds for supposing that climate had any curative influence. The most important point of agreement developed by the analysis of this group of cases relates to change of habits as regards exercise and out-door life, and the agreement in this respect is highly significant. III.—The third group embraces thirty-five cases. Only one of these cases was treated with tonic remedies, exclusive of cod-liver oil and alcoholic stimulants. In four tonics were employed in conjunction with alcoholic stimulants, and in two tonic remedies were conjoined with cod liver oil; alcoholic stimulants and cod liver oil were employed conjunctively in eight cases. Stimulants, oil, and tonics were used in one case. The curative remedies employed were only three in number: cod liver, alcoholic stimulants, and tonics of iron and quinine. In five of the thirty-five cases, the curative treatment consisted exclusively of cod-liver oil; in two of these the symptoms entirely ceased. Of these thirty-five cases, in fourteen the curative treatment consisted exclusively in the use of alcoholic stimulants; of these fourteen cases of arrest, in nine the recovery was apparently complete. Generous living was inculcated and adopted as far as practicable in all the cases.

The most striking and valuable of the results of the analytical study of these sixty-two cases is their almost uniform agreement as regards change of habits with respect to exercise and out-door life at the time of the arrest. Excluding the seven cases of the first class, and two in which the facts with respect to this point were not noted, of the remaining fifty-three, in all save three, the histories show a greater or less change of habits to have been made; and in many cases the change consisted in relinquishing sedentary callings for other pursuits, in order to carry out more effectually the desired reformation. Regarding the indications for the use of stimulants, Dr. Flint says:

"If their immediate effect be that of a cordial stimulant, that is, if they produce a sense of comfort; if they are followed by a feeling of increased strength, and a greater disposition to exercise; if they do not excite unduly the circulation or nervous system, I believe we may expect benefit from their use. *Per contra*, if their immediate effect be discomfort; if they are followed by a feeling of increased weakness and less disposition to exercise, and if they excite unduly the circulation or nervous system, I believe they will not do good, and may perhaps do harm."

With respect to the formation of habits of intemperance, he remarks: "In not one of the cases which I have reported has there been developed, so far as I know, a craving for stimulants, or a reliance upon them, rendering it difficult to relinquish their use. I have had my attention directed particularly to this point of observation, and I have not yet found an instance in which there was any apparent reluctance to discontinue the use of alcoholic stimulants whenever it was deemed advisable. I have not yet found an instance in which their use was continued after they were declared unnecessary; in short, up to this time I am not aware that in a single case among the many cases in which I have advised alcoholic stimulants, has a patient fallen into intemperate habits. * * * I certainly am not prepared to advocate the use of alcoholic stimulants as a prophylactic; that is, to sanction indulgence among those who may believe or fancy that they are in danger of becoming tuberculous. I would not advise their use in doubtful cases; they should follow a clear diagnosis, based on signs and symptoms. In persons with the unfortunate idiosyncrasy which leads to an irresistible craving on the slightest indulgence, the immediate effects would always contra-indicate their use in conformity with the rules which should govern our practice in cases of tuberculosis. And, finally, when employed as a remedy, they are not to be taken as a means of conviviality, or for any other than a curative influence."

Dr. Detmold was not able to reconcile the clinical and autopsical history of phthisis with the generally received theory as to its cause. It was claimed that the respiratory function was the one that suffered. This he could not believe, because, if there was a deficiency of oxygen admitted to the blood, digestion still remaining active, there would necessarily be a chance for the accumulation of facts. But the reverse of this was the case. Again, the most successful remedies used for phthisis were the hydrocarbons. This, to his mind, did not prove that the disease was due to any great want of oxygen.

Dr. Griscom thought that the disease depended on the lack of carbon in the blood instead of oxygen, and instanced in proof of that fact the beneficial influence of fats as remedial agents.

Dr. McCready alluded to the fact that alcohol was discharged from the body, as such, and acted beneficially only so far as it stimulated digestion and aided assimilation.

Dr. Horace Green had only employed alcohol as an adjuvant to other remedies, and therefore could only speak approximately of its effects.—Conjoined with tonics and out-door exercise, he had seen great benefit from its use.

Dr. Flint, jr., was of the opinion that inasmuch as the process of nutrition was very much interfered with, the indications were to bring that process to the normal state. He believed that alcohol conduced to such an effect, not by its actual consumption, but by its mere presence, the same as did common salt. He alluded in the course of his remarks to the results of experiments upon alcohol made by Surgeon-General Hammond, who claimed to prove that by its use the amount of urea excreted was really less than without it, and that a person could do more work with a less amount of food. With regard to the habitual practice of using ardent drinks, he remarked that it caused healthy persons to live too fast, and become in consequence prematurely old. In disease, however, the effect of alcohol was simply by its presence beneficial in stimulating digestion and aiding assimilation.

Dr. Parker spoke strongly against the practice of whiskey drinking, which was becoming so common in the community on the slightest pretence, and thought it well that practitioners should be on their guard against prescribing it when they were in any doubt concerning its beneficial effects.

Dr. Blakeman related the case of a young lady who, in consequence of the prescription of a physician, was led into habits of intemperance to such an extent that in the course of eight months she was accustomed to take two and a half pints of brandy daily. She died a drunkard.

Dr. Post related a similar case of a young man, with strong hereditary predisposition to consumption, who, upon the advice of a physician to stimulate freely, also became a drunkard, and eventually died of delirium tremens.

Dr. Peaslee was in favor of stimulants in phthisis, when a good effect could be obtained by nothing else. He considered alcohol useful as a stimulant to digestion and to the nervous system, as well as a generator of animal heat. He thought it impossible that alcohol should arrest retrograde metamorphosis, as claimed by Dr. Hammond.

Dr. Batchelder stated that during a practice of over half a century, having pretty generally prescribed stimulants, he had never known a single case of habitual drunkenness as the result.—*American Medical Times.*

